# The influence of life events on first and recurrent admissions in bipolar disorder

**DOI:** 10.1186/s40345-015-0022-4

**Published:** 2015-02-25

**Authors:** Sanne M Kemner, Neeltje EM van Haren, Florian Bootsman, Marinus JC Eijkemans, Ronald Vonk, Astrid C van der Schot, Willem A Nolen, Manon HJ Hillegers

**Affiliations:** Brain Center Rudolf Magnus, Department of Psychiatry, University Medical Center Utrecht, A00.241, PO Box 85500, 3508 GA Utrecht, The Netherlands; Julius Center for Health Sciences and Primary Care, University Medical Center Utrecht, Utrecht, The Netherlands; Reinier van Arkel Group, ’s-Hertogenbosch, The Netherlands; Alzheimer Nederland, Amersfoort, The Netherlands; Department of Psychiatry, University Medical Center Groningen, University of Groningen, Groningen, The Netherlands

**Keywords:** Bipolar disorder, Mood disorder, Life events, Twins, Admissions

## Abstract

**Background:**

Life events play an important role in the onset and course of bipolar disorder. We will test the influence of life events on first and recurrent admissions in bipolar disorder and their interaction to test the kindling hypothesis.

**Methods:**

We collected information about life events and admissions across the life span in 51 bipolar patients. We constructed four models to explore the decay of life event effects on admissions. To test their interaction, we used the Andersen-Gill model.

**Results:**

The relationship between life events and admissions was best described with a model in which the effects of life events gradually decayed by 25% per year. Both life event load and recurrent admissions significantly increased the risk of both first and subsequent admissions. No significant interaction between life event load and number of admissions was found.

**Conclusions:**

Life events increase the risk of both first and recurrent admissions in bipolar disorder. We found no significant interaction between life events and admissions, but the effect of life events on admissions decreases after the first admission which is in line with the kindling hypothesis.

## Background

The presence of psychopathology is often explained on the basis of stress-diathesis interactions (Monroe and Simons 1991). The diathesis-stress model serves to explore how non-biological or genetic traits (diatheses) interact with environmental influences (stressors) to trigger the onset of psychiatric disorders (Moffitt et al. 2005; Harris 2001). The environmental factor most frequently studied in this context is stress, often operationalized as life events. Numerous studies have demonstrated that life events play a role in the onset and course of both unipolar depression and bipolar disorder (Bender and Alloy 2011; Brown and Harris 1989; Hillegers et al. 2004; Hlastala et al. 2000; Malkoff-Schwartz et al. 1998).

Methodological limitations are a major issue when interpreting and comparing studies regarding the influence of life events on the onset and course of mood disorders (Johnson 2005). In many of these studies, data were obtained retrospectively, which complicates the reliable reporting of both life events and mood episodes due to recall bias. Moreover, regardless of the number of questions in an interview, people gradually forget life events (Paykel 1997; Brown and Harris 1982; Harris 2001). Furthermore, most studies so far used the number of episodes to define the course of illness whereas especially episodes longer ago are difficult to be remembered reliable, while it might be more reliable to report episodes which were associated with psychiatric admissions, as these are likely to reflect the most severe mood episodes and can often be confirmed with medical records.

The type of life event measures varies greatly between studies and poses another major obstacle in life event research. In particular, self-administered measures of stressful life events appear unreliable (Johnson 2005). Bender and Alloy (2011) confirmed that the gold standard of life stress measurements is the Life Events and Difficulties Schedule (LEDS) (Brown and Harris 1978). The LEDS provides the opportunity to categorize, date and rate both positive and negative life events. Furthermore, in contrast to life event questionnaires, the LEDS interview includes both major and minor types of stress, making it more suitable for testing the kindling hypothesis where life events play a greater role in the onset of initial episodes than in subsequent later episodes, which can even occur more or less spontaneously (Post 1992). The kindling model was originally described as the electrical kindling in relation to epilepsy where after many repetitions of kindled seizures ‘spontaneity’ occurs, i.e. seizures develop in the absence of external stimulation (Pinel 1981; Wada et al. 1974). Interestingly, several studies have demonstrated that a history of episodes is a significant risk factor for future recurrences in mood disorders (Judd et al. 2008; Keller et al. 1983; Perlis et al. 2006). In bipolar disorder, several studies report that after the first admission, 50% to 75% of patients have a recurrence within 4 to 5 years (Bromet et al. 2005; Leverich et al. 2001).

So far, research on the kindling hypothesis has mainly focused on unipolar depression and a majority of studies indeed found supportive evidence (Bender and Alloy 2011). However, studies in bipolar disorder are limited and findings are inconsistent. Bender and Alloy (2011) integrated the current literature and showed that about half the studies failed to find evidence for the kindling hypothesis in bipolar disorder. Crucially, LEDS interview-based studies all failed to find such evidence (Dienes et al. 2006; Hammen and Gitlin 1997; Hlastala et al. 2000; Swendsen et al. 1995). However, they did establish significant associations between the onset and course of bipolar disorder and life events.

In an ongoing naturalistic longitudinal twin study on bipolar disorder, we obtained detailed life event information throughout the life span by using the LEDS and were able to look for possible associations with first and recurrent admissions. Our aims are (1) to assess the influence of the effect of life events on first and recurrent admissions; (2) to assess the influence of prior admissions on the risk of subsequent admissions; and (3) to test the interaction between life event load and number of admissions (i.e. as indication for a kindling effect) in those twins with bipolar disorder.

## Methods

### Sample

We conducted a secondary analysis with three *a priori* questions within an ongoing study among twins (affected twin pairs, *n* = 51; healthy control twin pairs, *n* = 35) with bipolar disorder at the University Medical Center Utrecht (UMCU), The Netherlands. Of this cohort, all 51 twins with bipolar disorder (bipolar I disorder, *n* = 37; bipolar II disorder, *n* = 14) were included in the current study. The design of the study and the recruitment of the bipolar twin pairs have been described in detail elsewhere (Van der Schot et al. 2009; Vonk et al. 2007). All participants were enrolled between 2001 and 2006. There were no restrictions on duration or stage of illness for inclusion in the study, and all patients were treated naturalistically.

Demographic information is displayed in Table 1. All diagnoses were confirmed with the Structured Clinical Interview for DSM-IV (First et al. 1996) and the Structured Interview for DSM-IV Personality (Pfohl et al. 1997). Hospitalizations were confirmed through available medical records. Current mood state was assessed using the Young Mania Rating Scale (YMRS; (Young et al. 1978)) and the Inventory for Depressive Symptomatology (IDS; (Rush et al. 1996)). At the time of the study, all patients were euthymic with a YMRS score of 4 or less and an IDS score of 12 or less.

**Table 1:** **Demographics**

	**Total**	**Bipolar disorder type I**	**Bipolar disorder type II**
*n*	51	37	14
Female/male (*n*)	33/18	25/12	8/6
Age at LEDS interview, *M* (SD)	40.49 (9.52)	39.51 (8.71)	43.07 (11.33)
Age at onset of the first bipolar episode, *M* (SD)	28.20 (9.16)	26.19 (6.94)	33.50 (12.16)
Age at onset of the first symptoms (all), *M* (SD)	26.08 (8.85)	25.27 (6.82)	28.21 (12.86)
Age at onset of treatment, *M* (SD)	27.72 (9.03)	26.41 (7.71)	31.46 (11.59)
Comorbid disorder (1, 2 or 3), *n*(%)	11 (22%)	8 (22%)	3 (21%)
Psychotic symptoms lifetime, *n*(%)	26 (51%)	24 (65%)	2 (14%)
Hospitalized group			
Hospitalized patients, *n*(%)	35 (69%)	31 (84%)	4 (29%)
Number of admissions, *M* (SD)	3.06 (2.45)	2.93 (2.11)	4.00 (4.69)
Age at first admission, *M* (SD)	27.91 (7.86)	26.61 (6.4)	38 (11.83)
Type of episode at first admission (*n*)			
Mania	15	15	0
Depression	12	9	3
Psychosis	6	6	0
Others	2	1	1

The study was approved by the medical ethics review board of the University Medical Center Utrecht, and all participants gave written informed consent after full explanation of the study aims and procedures.

### Life event measures

All subjects included in the current study were interviewed with the investigator-based Bedford College LEDS (Brown and Harris 1978, 1989). The LEDS is a semi-structured interview for assessing life events and long-term difficulties in adults. It collects detailed information about the event itself, the timing of its occurrence (date) and relevant contextual information for each event. Each event is categorized into one of ten domains, consisting of education, work, reproduction, housing, money/possessions, crime/legal, health, marital/partner, other relationships and miscellaneous/death. Based on the contextual information, the threat for each event is rated via standardized rating procedures. The threat score represents the severity of the event, ranging from mild (1) to severe (4), hereby differentiating between mild life events and more stressful life events. The contextual threat is conceptualized as: ‘What most people would be expected to feel about an event in a particular set of circumstances and biography, taking no account of what the respondent says either about his or her reaction or about any psychiatric or physical symptoms that followed it’ (Brown and Harris 1989). Several studies have supported the reliability (e.g. interrater) and validity (e.g. multiple informant) of the LEDS in adults exhibiting a variety of psychiatric symptoms (Brown and Harris 1978, 1989; Ormel et al. 2001).

Only events occurring from the age of 5 years were included. All severe events were defined by the extent they were related to the bipolar disorder and to what extent they were dependent on the respondents’ own behaviour. To determine relatedness to the disorder, each severe event was rated on a three-point scale: 1) not related to psychopathology; 2) possibly related to psychopathology; or 3) clearly related to psychopathology. Only events with score 1 were included for further analyses. To determine if life events occurred independent of will or influence of the respondents’ own behaviour, each severe event was rated on a seven-point scale: 1) completely independent; 2) nearly independent; 3) possible influence, however, very unlikely; 4) physical illness; 5) cooperation or agreement with external situation; 6) likely neglect or carelessness; and 7) intentional choice. Events rating 1 to 5 were included for further analyses. Each life event was dated per year. Age was then calculated for each event.

All interviewers and raters were trained by MH, who was trained by G.W. Brown and T.O. Harris, who developed the LEDS. The interviews were conducted at the participant’s home or at the UMCU. Events were rated by two independent raters who had not been involved in the interviews. A panel consisting of the four raters (including SK and MH) reached consensus on the events that raised rating problems.

### Statistical analysis

#### Life event load

Life event load represents the sum of the threat scores of the life events occurring in each year.

We calculated three different life event load measures: (1) cumulative load (CL), i.e. the life event load at a particular point in time (year *Y*) calculated as the sum of the life event load in year *Y* and all preceding years; (2) cumulative load excluding events possibly or clearly related to the bipolar disorder (CL-NoBP); and (3) cumulative load including only independent events, thus excluding events possibly or clearly dependent on the respondents’ own behaviour (CL-I).

Next, the life event load before the first or since the last admission was calculated. After each admission, life event load was reset to zero and was calculated as described above. The cumulative life event load in the year preceding the admission was used for analysis.

#### Decay model

Previous studies showed a decay effect, implying that the presumed effect of life events diminishes over time, e.g. the death of a close relative that occurred 3 or 4 years before admission has less impact compared to the same event 1 year before admission (Hillegers et al. 2004). We will investigate which decay model statistically fits the data best. To explore the degree to which the effect of life events diminishes over time, a time-specific life event load variable was calculated for every year and subjected to an exponential decay function. We tested four models; in model I, we tested the purely cumulative effect, and in models II to IV, the decay function implied a 25%, 50% and 75% loss of effect per year, respectively. The decay function yielding the best model fit (−2× log-likelihood) will be used for all further analysis.

#### Andersen-Gill model

The Andersen-Gill model (A-G model), an extension of the standard Cox proportional hazard model for recurrent events, accommodates censored data and time-dependent covariates (Fleming and Harrington 1991; Therneau and Grambsch 2000).

Data for the A-G model are structured such that for each individual, intervals at risk are defined by variables describing the start and end times of each year of age. An event variable is coded as ‘1’ for admission and ‘0’ for no admission.

The A-G approach follows the usual assumption of the Cox model that the hazard or risk ratio is proportional over time and more specifically that the risk of being admitted is unaffected by earlier admissions. Time-dependent covariates, such as the cumulative load of life events or the number of previous admissions, may be used to relax the latter assumption. The hazard ratio represents the proportionate change in the ‘admission’ rate due to a unit change in the respective covariate, in this case the cumulative life event load.

#### Andersen-Gill model: interaction effect

The presence of an interaction effect will be tested by integrating an interaction function in the A-G model, testing the effect of the interaction between the number of admissions and the cumulative load between the admissions in the best-fitted decay model, also known as a kindling effect (Post 1992).

## Results

**Figure 1:**
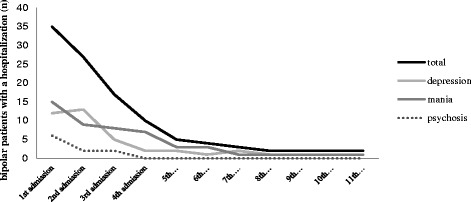
**Number and polarity of admissions.**

The general characteristics of our sample are shown in Table 1. At least one admission had occurred for 35 of the 51 bipolar patients, with a maximum of 11 admissions in two patients. Figure 1 and Table 2 display the number and polarity for all admissions.

**Table 2:** **Number of admissions**

**Total number of admissions**	***N*** **(total = 51)**
0	16
1	8
2	10
3	7
4	5
5	1
6	1
7	1
11	2

### Influence of life event effect on first and recurrent admissions

**Figure 2:**
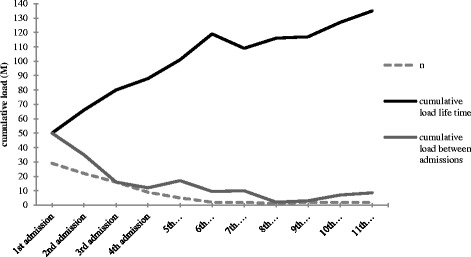
**Course of cumulative load.**

The relationship between life event load and admission (irrespective of the number of admissions) is depicted in Table 3. The exponentiated linear coefficients from the A-G model are interpreted as risk ratios relating the magnitude of a covariate (or multiple covariates) to admission. Positive coefficients indicate increased hazard for admission (‘1’ vs ‘0’). Figure 2 illustrates the cumulative life event load over time and the cumulative life event load between the admissions.

**Table 3:** **Relative risk of admission using four models of event effect decay**

**Model**	**Coefficient**	**Exp coefficient** ^**a**^	**Log-likelihood of fitted model**	***p***
Cumulative	.024	1.024	−308.5503	<.001
25% decay	.134	1.143	−293.5533^b^	<.001
50% decay	.240	1.272	−298.4862	<.001
75% decay	.452	1.572	−306.3194	<.001

^a^Exponentiated linear coefficients. ^b^Lowest absolute log-likelihood of fitted model.

Independent of the model employed (cumulative, 25%, 50% or 75% decay), the life event load was significantly associated with an increased risk of hospitalization per unit life event load. Adjustment for age and gender did not change the life event risk ratios. According to the log-likelihood, indicating the quality of fit, the decay model in which the life event load accumulates and at the same time decreases with a function of 25% with every subsequent year (model II) was most in agreement with the observed data. Therefore, all further analyses will be done under model II.

Table 4 displays the results of the A-G model with the three different types of load between the admissions: CL, CL-NoBP and CL-I.

**Table 4:** **Influence and interaction of types of cumulative load (25% decay), admissions and number of admissions**

	**Coefficient**	**Exp coefficient** ^**a**^	**SE (coef)**	**Robust SE** ^**b**^	***z***	***p***
Type of cumulative load between admissions						
CL	.086	1.09	.019	.021	4.17	<.001
Number of admissions	.560	1.75	.064	.093	6.03	<.001
CL-I	.093	1.10	.023	.021	4.35	<.001
Number of admissions	.577	1.78	.066	.099	5.81	<.001
CL-NoBP	.085	1.09	.027	.024	3.59	<.001
Number of admissions	.603	1.83	.069	.111	5.43	<.001
Interaction effect						
CL	.071	1.07	.027	.026	2.79	<.001
Number of admissions	.513	1.67	.091	.115	4.47	<.001
CL × number of admissions	.006	1.01	.009	.008	0.75	.45
CL-I	.081	1.08	.030	.023	3.49	<.001
Number of admissions	.537	1.71	.091	.118	4.56	<.001
CL-I × number of admissions	.006	1.01	.009	.010	0.58	.57
CL-NoBP	.053	1.05	.035	.024	2.25	<.05
Number of admissions	.510	1.67	.093	.125	4.07	<.001
CL-NoBP × number of admissions	.018	1.02	.012	.015	1.23	.22

CL, cumulative load including all events; CL-I, cumulative load including only independent events; CL-NoBP, cumulative load excluding events related to the disorder. ^a^Exponentiated coefficients, representing the hazard ratio. ^b^Robust standard error (SE), corrected for the dependency of multiple times to event within the same subject.

All coefficients for both life event load and number of admissions are positive and significant. Positive effect of all types of life event load indicates that the risk of getting admitted grows with an increasing life event load. This effect is independent of the type of life event load.

The A-G model with lifetime cumulative load and number of admissions shows a positive and significant risk ratio for both the lifetime load (coef = .0985, SE = .0166, *p* < .001) and number of admissions (coef = .4597, SE = .0892, *p* < .001), indicating that in addition to the cumulative load between the admissions, the lifetime cumulative load also contributes to the risk of getting admitted.

### Effect of number of previous admissions on admissions

The positive and significant coefficient of the number of admissions on the risk of getting admitted implies an increase in the chance of getting admitted with each subsequent admission.

### Interaction between life event load and number of admissions

The interaction effect between the cumulative load between admissions in model II (with 25% decay) and number of admissions did not reach significance, indicating that the effect of cumulative load on the risk of admission does not change with subsequent admissions. However, the influence of life events on first admissions is higher compared with the influence of life events after on readmissions, suggesting a shift in the effect of life events between the first and subsequent admissions.

Results did not change when excluding the concordant co-twins from the sample. Also, neither age, age of onset of the first bipolar episode, age of first admission nor gender affected any of the above findings.

## Discussion

Our main finding is that an increased life event load, taking into account the number and threat of life events, impacts both first and recurrent admissions in bipolar patients. This has also been found in previous studies (Bender and Alloy 2011; Hunt et al. 1992; Kessing et al. 1998, 2004), but it was hypothesized that this might be due to life events occurring as a consequence of the disease (Kessing et al. 2004). We now extended these previous findings by showing that the effect of life events on admissions did not change when events related to the disorder were excluded from the analyses. This suggests that the effect of life events is independent of life events occurring in relation to the disorder. We consider this robust influence of life events on first and recurrent admissions an important finding, as exposure and responses to life events are potentially modifiable. A better understanding of how they impact the risk of being admitted may yield specific strategies for prevention and early intervention.

Our next finding was that the effect of the number of prior admissions on the risk of getting admitted was positive and significant, demonstrating that the risk increases with each admission. Several studies reported that after the first admission for bipolar disorder, 50% to 75% of patients relapse within 4 to 5 years (Bromet et al. 2005; Leverich et al. 2001). Our findings indicate that the risk of readmission increases as a function of the number of previous admissions. Given our finding that the risk of getting admitted is independent of events that are related to the disorder, such as admissions, the association between the number of previous admissions and increased risk of readmission might be interpreted as an indicator for illness severity. Moreover, this finding also suggests a possible kindling effect; previous admissions could trigger the next admission.

Finally, we found no significant interaction between life event load and the number of prior admissions on the risk to be readmitted, suggesting that the effect of life event load does not decrease as a function of subsequent admissions. However, we did find a stronger effect of life events on the first compared to subsequent admissions which does suggest a possible kindling effect. In this respect, it should however be realized that the kindling effect has mostly been found after the occurrence of five to seven episodes (Kendler et al. 2000; Kendler and Gardner 2001; Slavich et al. 2011) while we only looked at admissions and the average number of admissions lies between three and four in our sample. Previous studies using the LEDS in bipolar patients (Dienes et al. 2006; Swendsen et al. 1995; Hammen and Gitlin 1997) looking at episodes rather than admissions did not find evidence for either presence or absence of a kindling effect. This is in contrast with findings in unipolar depression, which might be explained by the more complex course of contrasting mood episodes (i.e. mania and depression) in bipolar disorder as compared with unipolar depression (only depression). Bipolar episodes can be manic, hypomanic, depressive or mixed, and it is possible that the influence of life events differs across these various episodes.

**Figure 3:**
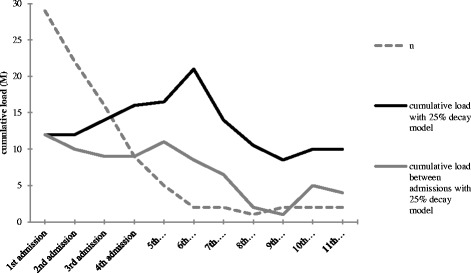
**Course of cumulative load under the 25% decay model.**

The effect of life events on admissions was best described by model II in which the influence of life events steadily accumulates (as one gets older, more life events occur) but at the same time gradually decays with 25% per year as time goes by (an event that has occurred years ago will no longer have the same impact as when it just happened). This decay model is in accordance with previous findings from our group in a sample of offspring of parents with bipolar disorder (Hillegers et al. 2004). The decay model of 25% (Figure 3) best explained the influence of stressful life events on the onset of mood disorders when compared to the purely cumulative model or models with 50% or 75% decay per year. The underlying mechanisms that cause this decay are not known; a possible explanation lies in the interaction of life stress with coping strategies and temperament. Coping responses influence the association between stress and the onset of mood episodes. Temperamental traits influence individual coping styles and modify the impact of stressful life events on mood episode onset (Compas et al. 2004).

There are several limitations that need to be taken into account when interpreting our findings. Firstly, methodological limitations are a major issue when interpreting and comparing studies regarding the influence of life events on the onset and course of mood disorders (Johnson 2005). In many of these studies, information on life events was obtained retrospectively with queries or (semi-) structured interviews, which complicates the reliable reporting due to recall bias. Regardless of the number of queries in an interview, people gradually forget life events (Paykel 1997; Brown and Harris 1982; Harris 2001). The average participant in our sample had to report life events over a time span of 35 years. One could question the reliability of the LEDS when it is used retrospectively to collect lifetime life event data. Most studies restrict the reporting of life events to a 12-month period. However, the LEDS is probably more reliable compared to (retrospective) checklist inventories (Hillegers et al. 2004; Ormel et al. 2001), as the LEDS minimizes recall bias; information is actively obtained in a very structured interview by detailed questions in ten domains. Furthermore, there is evidence that recall bias is more pronounced for minor events, suggesting that major life changes are under less influence of recall bias (Funch and Marshall 1984).

Secondly, more than one admission could occur per year. It is not clear to what extent this influenced our results, since admissions occurring within 3 to 6 months after the first admission are associated with more subclinical affective symptoms and therefore could be due to the same bipolar episode (Bromet et al. 2005). Unfortunately, the data on life events was dated per year and did not allow us to conduct the analysis in more detail.

We made no distinction between admissions due to mania, depression or psychosis. However, as can be seen in Figure 1, the polarity of the admissions is equally divided across the number of admissions for manic and depressive episodes.

Our sample is drawn from a longitudinal twin study. Having participants in the sample that share their genes and environment to a large extent might influence the study results. However, excluding the bipolar co-twins (*n* = 8) resulting in only one twin per pair in the analysis (*n* = 43) did not change our findings.

Finally, although most analyses yielded significant results, we have a small sample size consisting of patients with bipolar I as well as bipolar II disorders. So far, most studies limit their sample to bipolar type I (Bender and Alloy 2011). The small sample size did not allow us to compare the two subtypes.

## Conclusions

Life events, taking into account the number and threat of life events, appeared to have an impact on both first and recurrent admissions in bipolar patients, and this effect appeared not be dependent on events related to the illness. In addition, the number of prior admissions was positively related to the risk of getting readmitted. Finally, we did not find an interaction between life events and admissions on the risk for readmission, although the effect of life events was stronger on first admissions compared to readmissions, which suggests a possible kindling effect.

## Abbreviations

A-G model: Andersen-Gill model
CL: cumulative load
CL-I: cumulative load including only events occurring independent of the respondents’ own behaviour
CL-NoBP: cumulative load excluding all events occurring as a consequence of bipolar disorder
LEDS: Life Events and Difficulties Schedule
